# Expected Value of Sample Information to Guide the Design of Group Sequential Clinical Trials

**DOI:** 10.1177/0272989X211045036

**Published:** 2021-12-03

**Authors:** Laura Flight, Steven Julious, Alan Brennan, Susan Todd

**Affiliations:** School of Health and Related Research, University of Sheffield, Sheffield, UK; School of Health and Related Research, University of Sheffield, Sheffield, UK; School of Health and Related Research, University of Sheffield, Sheffield, UK; Department of Mathematics and Statistics, University of Reading, Reading, UK

**Keywords:** adaptive designs, clinical trials, expected value of sample information, bias adjustment, value of information analysis

## Abstract

**Introduction:**

Adaptive designs allow changes to an ongoing trial based on prespecified early examinations of accrued data. Opportunities are potentially being missed to incorporate health economic considerations into the design of these studies.

**Methods:**

We describe how to estimate the expected value of sample information for group sequential design adaptive trials. We operationalize this approach in a hypothetical case study using data from a pilot trial. We report the expected value of sample information and expected net benefit of sampling results for 5 design options for the future full-scale trial including the fixed-sample-size design and the group sequential design using either the Pocock stopping rule or the O’Brien-Fleming stopping rule with 2 or 5 analyses. We considered 2 scenarios relating to 1) using the cost-effectiveness model with a traditional approach to the health economic analysis and 2) adjusting the cost-effectiveness analysis to incorporate the bias-adjusted maximum likelihood estimates of trial outcomes to account for the bias that can be generated in adaptive trials.

**Results:**

The case study demonstrated that the methods developed could be successfully applied in practice. The results showed that the O’Brien-Fleming stopping rule with 2 analyses was the most efficient design with the highest expected net benefit of sampling in the case study.

**Conclusions:**

Cost-effectiveness considerations are unavoidable in budget-constrained, publicly funded health care systems, and adaptive designs can provide an alternative to costly fixed-sample-size designs. We recommend that when planning a clinical trial, expected value of sample information methods be used to compare possible adaptive and nonadaptive trial designs, with appropriate adjustment, to help justify the choice of design characteristics and ensure the cost-effective use of research funding.

**Highlights:**

- Opportunities are potentially being missed to incorporate health economic considerations into the design of adaptive clinical trials.
- Existing expected value of sample information analysis methods can be extended to compare possible group sequential and nonadaptive trial designs when planning a clinical trial.
- We recommend that adjusted analyses be presented to control for the potential impact of the adaptive designs and to maintain the accuracy of the calculations.
- This approach can help to justify the choice of design characteristics and ensure the cost-effective use of limited research funding.

Conducting efficient research is a priority for funders with limited heath research budgets.^1^ Adaptive designs are one way to potentially make a trial more efficient that use data collected as a trial progresses, at prespecified time points, to inform modifications to the trial.^2^ They can directly benefit patients and health care providers both ethically and financially.^3,4^ Adaptive designs are increasingly used^5–7^ and have been adopted in trials for the treatment of COVID-19.^8^

The methods of value-of-information analysis (VOIA) provide a framework for quantifying the value of collecting more information to determine whether a treatment should be adopted by balancing the benefits of additional research against the costs. To date, opportunities are potentially being missed to apply VOIA methods into the design and analysis of adaptive designs.^9,10^

In this article, we extend existing VOIA methods, specifically the expected value of sample information (EVSI) for assessing the cost-effectiveness of proposed fixed-sample-size designs to the adaptive design setting.^11^ This approach helps to increase the efficiency of trials while reflecting stakeholder preferences for adaptive decisions to be informed by clinical effectiveness during the trial.^11^ We highlight how this approach differs from the fixed-sample-size design setting using the ISPOR Value of Information Emerging Good Practices Task Force framework.^10,12^ Key considerations include appropriately adjusting estimates for the adaptive nature of the design as well as capturing the costs associated with conducting an adaptive design.

Using a hypothetical case study, based on the CACTUS pilot trial,^13^ the methods are used to guide the design of a trial focusing on the number of interim analyses and choice of clinical effectiveness stopping rule while making appropriate adjustments for the adaptive nature of the design.

## Group Sequential Designs

Pallmann et al.^4^ provided a summary of available adaptive designs. In this article, we focus on the commonly used group sequential designs (GSDs).^5,14^ During a GSD, data are examined multiple times. At an interim analysis, a test statistic comparing the intervention and control arms for the outcome of interest—typically, a clinical rather than a cost-effectiveness outcome—is calculated. This test statistic is then compared with the prespecified stopping boundary (also known as a stopping rule).

If the test statistic falls within the boundary, the trial continues to the next analysis. This process is repeated at each interim analysis using all accumulated evidence until the test statistics cross the boundary or reach the final analysis. Examples of stopping rules include those proposed by Pocock^15^ and O’Brien-Fleming.^16^ Each rule has different characteristics and impact on the design and subsequent analyses.^17^ The Pocock rule requires a larger maximum sample size if the trial does not stop early; however, there is a lower hurdle for stopping the trial at early analyses compared with the O’Brien-Fleming rule.

It is important to account for the adaptive nature of the design during analysis to avoid introducing bias into the trial results.^14^ Flight^18^ explored the impact that a GSD can have on the health economic analysis following a clinical trial and showed how it is important to adjust for the adaptive design to maintain an accurate health economic analysis.

In this article, we consider the appropriate adjustment for the adaptive nature of the trial when extending VOIA methods to guide the design of GSDs, highlighting the additional considerations in the adaptive design setting. Our approach allows researchers to determine a cost-effective design by comparing different stopping rules and number of interim analyses and to compare adaptive with fixed-sample-size designs.

## Methods

To conduct a VOIA for a fixed-sample-size design, Fenwick et al.^12^ proposed 7 steps in the ISPOR Value of Information Emerging Good Practices Task Force guidance. The following sections discuss each of these steps in the context of employing an adaptive design, highlighting how they differ from the fixed-sample-size design case. Here, step 6 has been modified for adaptive designs.

This work was supported by a public advisory group who ensured that the development of the methods was relevant and appropriate from the public perspective. More information on their role is provided by Flight.^18^

### Steps 1 and 2: Conceptualize and Construct a Health Economic Model and Parametrize with Evidence

As for fixed-sample-size designs, a health economic model needs to be constructed for the population of interest. This may be an existing model that has been developed for the disease of interest or from previous work such as a pilot study.

### Steps 3 and 4: Generate the Probabilistic Sensitivity Analysis Sample and Identify Uncertainty

A probabilistic sensitivity analysis (PSA) is generated based on available prior evidence for the model parameters, denoted by θ . Model parameters might include transition probabilities, costs, and benefits for a health state. We denote the PSA sample by

(1) $$\left\{\theta^{\left(1\right)},\ldots ,\theta^{\left(N_{\mathrm{PSA}}\right)}\right\}$$

where N_PSA_ is the number of PSA samples. For each row of the PSA sample, the model is evaluated to give a per-person net benefit for each intervention, denoted by

(2) $$\left\{\mathrm{NB}(d,\theta^{\left(1\right)}),\ldots ,\mathrm{NB}(d,\theta^{\left(N_{\mathrm{PSA}}\right)})\right\}$$

where *d* represents the interventions. There is no difference between the VOIA approach for a fixed-sample-size and adaptive design at this stage.

### Step 5: Establish whether Further Research is Worthwhile

The expected value of perfect information (EVPI) considers the scenario in which further research would eliminate all decision uncertainty.^19–21^ Further research is potentially worthwhile if the associated costs are less than the EVPI.^22^ Using the same approach as for a fixed-sample-size design, this can be calculated for the adaptive design using^19^

(3) $$\mathrm{EVPI}=E_{\theta }\left\{\underset{d}{\max}\mathrm{NB}(d,\theta )\right\}-\left\{\underset{d}{\max}E_{\theta }\left(\mathrm{NB}\left(d,\theta \right)\right)\right\}.$$

It may be possible to resolve all the uncertainty about a subset of the model parameters.^10^ This can be measured using the expected value of partially perfect information (EVPPI).^20^

### Step 6: Estimate the Value of Specific Research

If the EVPI calculation suggests further research is worthwhile, the value of different research designs can be estimated by calculating the EVSI and expected net benefit of sampling (ENBS). At this stage, additional considerations are required for an adaptive design. We break this step down into 6 stages:

1. Identify the trial designs for comparison.
2. Simulate the trial results and analysis data sets.
3. Calculate summary statistics adjusting the point estimates and confidence intervals of primary and secondary endpoints to allow for the adaptive nature of the trial design.
4. Calculate the EVSI.
5. Calculate the cost of sampling, accounting for any additional and potential costs savings.
6. Compare the ENBS of the proposed trial designs.

#### Stage 1: Identify the trial designs for comparison

The first stage is to choose the trial designs for consideration, which includes the sample size for the trial and the criteria on which the trial might stop early (the stopping rule). As discussed by Flight et al.,^9^ this is typically informed by the clinical primary outcome, as cost-effectiveness outcomes are rarely used in the design of an adaptive trial. As with the fixed-sample-size design, and using a frequentist approach to sample size calculation, this will require an estimate of the clinically important difference for the primary outcome, an estimate of the population variance (for a normal outcome) and type I and type II errors (typically chosen to be 0.05 and 0.1, respectively).^23^ These choices are the same regardless of the adaptive nature of the trial and are usually informed by prior information or discussions with the clinical research team.

Additional considerations for an adaptive design—specifically, a GSD here—include the choice of stopping rule (based on the clinical primary outcome) and the number of interim analyses. We consider GSDs with the Pocock stopping rule and the O’Brien-Fleming stopping rule with up to 5 equally spaced analyses of the data. The sample size is informed by these choices.

#### Stage 2: Simulate the trial results and analysis data sets accounting for the adaptive design

A trial result data set representative of the population to be randomized into the future trial is simulated for each row of the PSA sample. This is based on the likelihood function from existing information such as a pilot study or observational study. Rothery et al.^10^ suggested that data sets should be simulated taking into account how the data from the trial would be analyzed. Flight^18^ showed that the adaptive nature of a trial can affect the subsequent health economic analysis. Failing to adjust for this could result in a spurious estimate of the EVSI, potentially wasting limited resources. They also describe how bias-adjusted maximum likelihood estimate methods for the adjustment of the point estimate and the sample mean ordering approach to calculate adjusted confidence intervals of primary and health economic outcomes can be extended to adjust a within-trial and model-based health economic analysis.^24–26^

In this article, the bias-adjusted methods are referred to as the “adjusted analysis,” and the usual maximum likelihood estimate is referred to as the “unadjusted analysis.”

The data simulation is informed by the PSA parameter estimates to give a trial analysis data set in each row of the PSA. The trial analysis for the design under consideration is applied to each trial results data set. For an adaptive design, this establishes whether the trial would have stopped early at any of the interim analyses. For example, the first group of simulated participants form the analysis set at the first interim analysis. The primary outcome is calculated and compared with the prespecified stopping boundary. If the estimate crosses the boundary, the trial stops and the trial analysis data set is formed from the participants randomized into the trial up to that point. If the boundary is not crossed, the trial continues to the next interim analysis, until the point estimate crosses the boundary or the final analysis is reached. This is repeated for each row of the PSA sample. The accumulating cost-effectiveness data are not used to inform whether the trial should stop early.

#### Stage 3: Calculate summary statistics

Summary statistics for primary and secondary outcomes informing the health economic model are estimated from the trial analysis data set in each row of the PSA sample. This will include the primary and secondary clinical outcomes and health economic outcomes, such as health care resource use and health-related quality of life. These statistics are denoted by

(4) $$\left\{\tilde{T}(y^{\left(1\right)}),\ldots ,\tilde{T}(y^{\left(N_{\mathrm{PSA}}\right)})\right\},$$

for the adjusted analysis and by

(5) $$\left\{\hat{T}(y^{\left(1\right)}),\ldots ,\hat{T}(y^{\left(N_{\mathrm{PSA}}\right)})\right\},$$

for the unadjusted analysis.

#### Stage 4: Calculate the EVSI

The EVSI is the difference between the expected net benefit given sample information minus the expected net benefit given current information. The health economic model has input parameters (θ) to estimate the net benefit of each intervention ( d=1,…,D ) under consideration. This gives a per-person EVSI of

(6) $$\mathrm{EVSI}=E_{Y}\left\{\underset{d}{\max}E_{\theta |Y}\mathrm{NB}(d,\theta )\right\}-\left\{\underset{d}{\max}E_{\theta }\left(\mathrm{NB}\left(d,\theta \right)\right)\right\},$$

for data *Y* to be collected.^27^ A population-level EVSI is estimated by multiplying the individual-level EVSI by the time horizon ( T ) and the annual prevalence for the population ( $N_{p}$ ) to give^28^

(7) $$\mathrm{popEVSI}\;=\;\mathrm{EVSI}\times T\;\times \;N_{p}.$$

Methods for efficiently calculating the EVSI are summarized by Health et al.^29^ and Kunst et al.^30^ We use the nonparametric regression approach, as this does not require the existence of conjugate distributions or parametric assumptions.^27^

#### Stage 5: Calculate the cost of sampling accounting for additional costs and cost savings

We need to understand the costs associated with conducting the research, known as the cost of sampling.^31^ The total cost of sampling is composed of fixed, variable, analysis, and opportunity costs and depends on the number of participants recruited and number of analyses conducted. Fixed costs are incurred regardless of the trial design and include site recruitment and training, archiving costs, and dissemination. Variable costs include randomizing and following up participants such as staff costs and database management. The analysis costs include costs associated with conducting an analysis of the endpoints used to inform interim decision making (typically clinical endpoints). For the fixed design, the cost of analysis is included in the fixed costs. For the adaptive design, however, we separate this out, as multiple analyses may take place depending on the design chosen.

The opportunity cost can be thought of as the financial cost of delaying a decision to obtain more information.^32^ Willan and Kowgier^33^ suggested, for a 2-arm trial, the opportunity cost is equal to the incremental net benefit (INB) of the new intervention compared with the control based on information available before the trial begins.

The cost of sampling, for a 2-arm trial, is calculated using

(8) $$\mathrm{TC}=C_{f}+N_{a}C_{a}+nC_{v}+n_{I}C_{v,I}+n_{C}C_{v,C}+\frac{n}{2}C_{o},$$

where *TC* is the total cost of sampling, $C_{f}$ is the fixed cost, $N_{a}$ is the expected number of analyses, *$C_{a}$* is the cost of analysis, n is the expected sample size (ESS), *$C_{v}$* is the variable cost per patient incurred by every participant in the trial, *$n_{I}$* is the expected number of participants in the intervention arm, *$C_{v,I}$* is the variable cost per participant incurred in the intervention arm only, *$n_{C}$* is the expected number of participants in the control arm, $C_{v,C}$ is the variable cost per participant incurred in the control arm only, and $C_{o}$ is the opportunity cost per participant. Additional information on how to calculate the cost of sampling for an adaptive design is given in the supplementary material.

#### Stage 6 and Step 7: Compare the ENBS of trial designs and iterate with new evidence

The ENBS is the difference between the population EVSI and the cost of sampling. This can be calculated using the adjusted approach, denoted by $\tilde{\mathrm{ENBS}}$ , or the unadjusted approach, denoted by $\hat{\mathrm{ENBS}}$ . The optimal design from a health economic perspective has the highest ENBS. We use the ENBS to guide the design of a clinical trial alongside discussions with clinical teams, including the use of adaptive as well as fixed-sample-size designs. As for the fixed-sample-size design, this process should be repeated once new evidence is available.

The steps for conducting a VOIA for a fixed-sample-size design, proposed by Fenwick et al.,^12^ have been extended for an adaptive design. These methods appropriately adjust the analysis for the adaptive nature of the trial and capture the potential additional costs and cost savings of these designs. The following sections outline a hypothetical case study used to illustrate the approach and summarize the results.

### Hypothetical Case Study

We use a hypothetical case study, based on a real trial, to illustrate how VOIA can be applied to an adaptive design. The Cost-effectiveness of Aphasia Computer Treatment Compared to Usual Stimulation (CACTUS) pilot clinical trial aimed to assess the feasibility of conducting a large-scale clinical trial into the effectiveness of self-managed computer treatment for people with long-standing aphasia post stroke.^12^ Participants were randomized to either receive a computer-based intervention (CSLT) designed to improve word-finding ability through language exercises or a usual care control (UC). A model-based cost-utility analysis of pilot data provided an early analysis of the likely cost-effectiveness of CSLT, and full details are reported by Latimer et al.^34^

We considered alternative designs for a full-scale clinical trial following the CACTUS pilot comparing CSLT and UC. Using R, we adapted the original model and analysis methods reported by Latimer et al,^34^ and the proposed designs did not attempt to replicate the Big CACTUS clinical trial or health economic analysis that followed the CACTUS pilot trial.^35^ Full details on the economic model used in this analysis are provided by Flight.^18^

#### Trial design and data characteristics

We compared a fixed-sample-size design, Pocock (POC)^15^ and O’Brien-Fleming (OBF)^16^ stopping rules with maximums of 2 and 5 analyses. Each design was applied in R (version 3.4.3) using the RCTdesign package (http://www.rctdesign.org/Welcome.html). For each design, the type I and type II error rates were 0.05 and 0.1, respectively. The clinically important difference was the improvement in proportion of words named correctly between the intervention and control arm (treated as a continuous variable). That and its associated standard deviation were calculated using the pilot trial data.

To explore the impact of different trial designs and data characteristics on the choice of optimal design, the correlation between primary and health economic outcomes and the intervention costs were varied. We assumed there was a negative correlation between the primary outcome and costs and a positive correlation between the primary outcome and utilities. Absolute correlations of 0.0, 0.4, and 0.8 were explored, covering a range of no, medium, and high correlation. The cost of CSLT was varied over 15 values, and the INB from the pilot trial and the subsequent EVSI and ENBS was recalculated.

#### Data-generating mechanism

To generate the PSA sample, we bootstrapped the CACTUS pilot data 5000 times. We simulated a trial result data set for each of the PSA rows using copulas. This allowed the marginal distributions of the primary and the health economic outcomes (resource costs and utility) to be nonnormal and correlated. Full details are provided in the supplementary material. We used a willingness-to-pay threshold of £20,000 per quality-adjusted life-year as per National Institute for Health and Care Excellence guidance with a discount rate of 3.5% applied to costs and benefits.^36^ The time horizon and prevalent population were taken from the Latimer et al.^34^ pilot health economic analysis, giving an average of 27,616 patients expected to eligible for and compliant with CSLT per year over a 10-y period.

#### Trial results estimates

We calculated adjusted and unadjusted estimates of the health economic model parameters using the bias-adjusted maximum likelihood estimates described by Flight,^17^ compared adjusted and unadjusted point estimates of the primary clinical outcome, and reported the width of the 95% confidence interval. We then calculated and compared the ENBS for each of the scenarios to determine the optimal trial design from a health economic perspective.

## Results

We summarize how the results of such analyses could be presented when exploring the optimal trial design, including both fixed-sample-size and adaptive designs, by first considering the impact of each design on the maximum and ESS for the trial, the difference in the cost of sampling, and how the EVSI and ENBS for the designs might be compared visually and summarizing the potential impact of the unadjusted versus the adjusted approaches. FIX denotes the fixed-sample-size design, and OBF2 and OBF5 and POC2 and POC5 denote the O’Brien-Fleming and Pocock designs with 2 and 5 analyses, respectively. The specific results from applying the new VOIA methods to the hypothetical case study are context dependent and not generalizable to all VOIA calculations using this approach.

### Maximum and ESS and Proportion of Trials Stopping at Each Analysis

Table 1 summarizes the ESS, number of analyses, and distribution of the sample size for each design over the interim and final analyses with zero correlation between the primary and health economic outcomes. OBF5 has the highest expected number of analyses (4.55 analyses) and POC2 the fewest (1.79 analyses). Both POC designs have a high maximum sample size because of the large penalty for early examinations of the data. The ESS for these designs is high, as a large proportion of trials reach the final analysis where the sample size is larger than the fixed-sample-size design. A small number of trials (0.02%) stopped at the first interim analysis of OBF5, based on the accumulating evidence for the primary outcome, where the sample size was 60. In contrast, almost 5% of trials stopped at the first analysis of POC5, where the sample size was 72.

**Table 1:** Expected Number of Analyses, Expected Sample Size, Proportion of Trials Stopping at Each Analysis, and Expected Cost of Sampling for 5000 Probabilistic Sensitivity Analysis Samples, Assuming Zero Correlation between the Primary and Health Economic Outcomes

Design	Analysis	FIX	OBF 2	OBF 5	POC 2	POC 5
Expected number of analyses		1.00	1.91	4.55	1.79	4.28
Maximum sample size		292	294	300	320	352
Expected sample size		292.00	280.66	273.05	285.73	301.52
Proportion of simulated trials stopping at each analysis (expected No. of participants at each analysis)	1	1.00 (292)	0.09 (148)	0.00 (60)	0.21 (160)	0.05 (72)
	2	—	0.91 (294)	0.03 (120)	0.79 (320)	0.09 (142)
	3	—	—	0.11 (180)	—	0.09 (212)
	4	—	—	0.13 (240)	—	0.07 (282)
	5	—	—	0.73 (300)	—	0.70 (352)
Expected cost sampling (£million)		2.13	2.07	2.04	2.10	2.18
Expected cost of sampling for a trial stopping at each analysis (£ million)	1	2.13	1.42	0.98	1.48	1.04
	2	—	2.14	1.28	2.27	1.39
	3	—	—	1.57	—	1.73
	4	—	—	1.87	—	2.08
	5	—	—	2.17	—	2.42

OBF, O’Brien-Fleming stopping rule; POC, Pocock stopping rule.

### Calculating the Cost of Sampling for the Case Study

Financial information from the Big CACTUS grant application (not the actual costs incurred) was used to inform the cost of sampling for the hypothetical CACTUS case study. These detailed costs are routinely outlined in the planning of clinical trials and are a useful source for any trial team considering this approach. The components of the cost of sampling for the hypothetical case study are given in the supplementary material.

The cost of sampling for each design is given in Table 1. The cost of sampling for FIX is £2,127,530, the highest cost of sampling of the 5 designs. POC2, OBF2, and OBF5 have similar costs of sampling because of their similar ESS. POC5 has the highest costs of sampling; however, this is only £140,000 greater than the cheapest design (OBF5), which is relatively small given each design has a cost of sampling greater than £2,000,000. Even when a trial can stop at the first analysis, large costs are incurred, especially when the first analysis is conducted halfway through the trial. The trials stopping at the first analysis of 5 have the smallest cost of sampling, as they have one-fifth of the maximum number of participants. This is slightly smaller for OBF as the first analysis is conducted on the fewest number of participants.

### EVSI and ENBS

Table 2 gives the unadjusted EVSI and ENBS for FIX, as there are no early examinations of the data, there is no need to adjust the final analysis. The adjusted EVSI and ENBS are presented for the 4 adaptive designs to reflect the adjustments required.

**Table 2:** Results for 5 Proposed Trial Designs under 3 Different Scenarios for the Extent of Correlation between Primary and Health Economic Outcomes, with Correlation 0.0, 0.4, and 0.8. Based on 5000 Probabilistic Sensitivity Analysis Samples^a^

Design	FIX	OBF 2	OBF 5	POC 2	POC 5
Correlation = 0.0					
EVSI per patient (SE)	26.62 (5.17)	**27.27 (5.60)**	26.20 (5.41)	25.83 (5.49)	25.13 (5.41)
Population EVSI (million)	7.35	**7.53**	7.23	7.13	6.94
ENBS (£ million)	5.22	**5.46**	5.20	5.04	4.76
Correlation = 0.4					
EVSI per patient (SE)	39.91 (6.12)	**40.56 (6.32)**	38.84 (6.05)	37.99 (5.83)	40.91 (6.43)
Population EVSI (million)	11.02	**11.20**	10.73	10.49	11.30
ENBS (£ million)	8.89	**9.13**	8.69	8.39	9.12
Correlation = 0.8					
EVSI per patient (SE)	36.25 (5.83)	**40.13 (6.08)**	37.95 (6.12)	37.68 (5.92)	38.56 (5.99)
Population EVSI (million)	10.01	**11.08**	10.48	10.41	10.65
ENBS (£ million)	7.88	**9.01**	8.44	8.31	8.47

ENBS, expected net benefit of sampling; EVSI, expected value of sample information; OBF O’Brien-Fleming stopping rule; POC, Pocock stopping rule.

a Unadjusted values are presented for FIX and adjusted values for the adaptive designs. Values in **bold** show the most efficient design.

OBF2 has the highest EVSI and ENBS. From a cost-effectiveness perspective, this is the optimal trial design. This design gives a high EVSI but incurs a smaller cost of sampling compared with FIX and POC (see Table 1). The saving in costs of the additional, earlier analyses of OBF5 do not outweigh the reduction in EVSI as a result of the smaller ESS. Likewise, both POC designs do not perform well, as only a small number of trials stop early, and so the trial has a large cost of sampling with no gain in EVSI.

The 95% confidence intervals for the estimated EVSI are wide and overlapping for all scenarios. Increasing the number of PSA samples may reduce the variance; however, this will need to be balanced against the increased computation time. As this estimate is used to calculate the ENBS, the choice of design for the trial is uncertain if considering the EVSI and ENBS for 5000 PSA samples.

### Varying Intervention Costs in the Case Study

**Figure 1:**
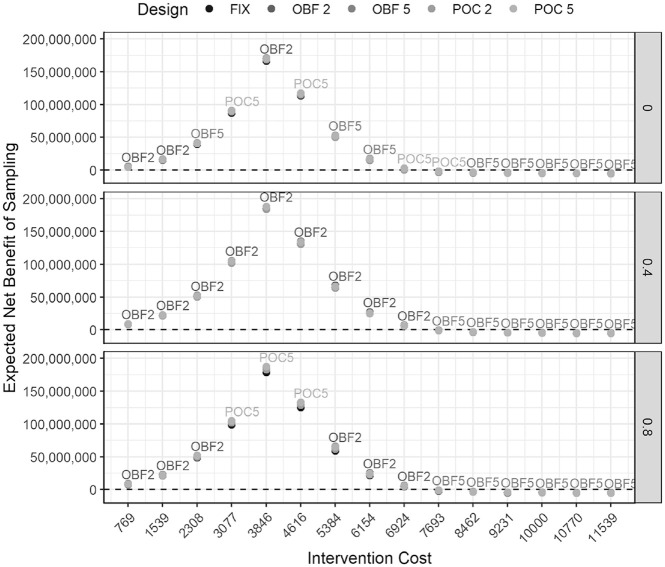
Case study sensitivity to the intervention cost assumption. Expected net benefit of sampling (ENBS) for 5 designs (5000 PSA samples). The adjusted ENBS is presented for the adaptive designs and the unadjusted ENBS for the fixed-sample-size design.

Figure 1 summarizes the ENBS for the 5 proposed designs for increasing intervention costs. This provides a useful way to visualize and compare the competing trial designs to identify the optimal option under increasing intervention costs, identifying scenarios in which different designs may be optimal. In the hypothetical case study, the OBF2 design performs best for all correlations when the intervention costs are low, as they have a higher EVSI and low cost of sampling. The POC5 design also performs well for lower intervention costs, especially when correlations are equal to zero and 0.8, as it has a high EVSI that outweighs its high cost of sampling. However, once the intervention costs are higher than approximately £8000 the designs with the smaller cost of sampling are preferable because of the small EVSI gained from all designs. Hence, the designs with 5 analyses perform better.

The ENBS increases as the intervention cost increases, as there is greater uncertainty in the cost-effectiveness decision. Once the intervention costs reach £3846, this uncertainty decreases, as it becomes clearer that the CSLT is unlikely to be cost-effective. POC5 performs well when there is highest uncertainty, as this has the highest ESS and thus the opportunity to learn more information from more participants.

### Comparison of Adjusted and Unadjusted EVSI

Table 3 summarizes the adjusted and unadjusted estimates of the EVSI, ENBS, health economic model parameters, and the primary outcome from the PSA samples. There is no difference between estimates for the baseline utility in the control arm, as this is not affected by the design of the trial and is thus set to be equal.

**Table 3:** Adjusted and Unadjusted Estimates of the EVSI, ENBS, and their Percentage Differences for Health Economic Model Parameters and the Clinical Primary Outcome

Design		FIX	OBF 2	OBF 5	POC 2	POC 5	FIX	OBF 2	OBF 5	POC 2	POC 5	FIX	OBF2	OBF 5	POC 2	POC 5
Correlation		0.0					0.4					0.8				
EVSI	Adjusted	—	27.3	26.2	25.8	25.1	—	40.6	38.8	38.0	40.9	—	40.1	37.9	37.7	38.6
	Unadjusted	26.6	25.8	24.4	24.1	23.8	39.9	37.2	35.2	36.0	36.9	36.2	35.4	34.9	34.5	35.9
	% Difference	—	5.6	7.3	6.8	5.4	—	8.7	9.8	5.4	10.3	—	12.5	8.4	8.8	7.2
ENBS	Adjusted	—	5.5	5.2	5.0	4.8	—	9.1	8.7	8.4	9.1	—	9.0	8.4	8.3	8.5
	Unadjusted	5.2	5.0	4.7	4.6	4.4	8.9	8.2	7.7	7.8	8.0	7.9	7.7	7.6	7.4	7.7
	% Difference	—	7.8	10.3	9.8	8.0	—	10.8	12.2	6.9	12.9	—	15.6	10.5	11.2	9.1
Resource cost intervention arm	Adjusted	—	201.4	201.4	201.6	201.8	—	201.9	201.9	202.1	202.6	—	202.3	202.5	202.4	202.8
	Unadjusted	201.5	201.4	201.4	201.6	201.8	201.6	201.4	201.4	201.4	201.1	202.2	201.9	201.9	201.8	201.6
	% Difference	—	0.0	0.0	0.0	0.0	—	0.2	0.2	0.3	0.7	—	0.2	0.3	0.3	0.6
Resource cost control arm	Adjusted	—	269.8	269.7	269.7	269.6	—	269.6	269.5	269.5	269.1	—	269.4	269.3	269.3	268.9
	Unadjusted	269.8	269.8	269.7	269.6	269.5	269.9	270.1	270.2	270.1	270.5	269.8	269.8	269.9	269.8	270.2
	% Difference	—	0.0	0.0	0.0	0.0	—	–0.2	–0.3	–0.2	–0.5	—	–0.2	–0.2	–0.2	–0.5
Utility improvement	Adjusted	—	0.0	0.0	0.0	0.0	—	0.3	0.3	0.3	0.3	—	0.2	0.2	0.2	0.2
	Unadjusted	0.0	0.0	0.0	0.0	0.0	0.3	0.3	0.3	0.3	0.3	0.2	0.2	0.2	0.2	0.2
	% Difference	—	–0.3	–0.5	–0.4	–1.0	—	–1.0	–1.5	–1.4	–3.1	—	–1.0	–1.5	–1.4	–3.1
Probability of a good response	Adjusted	—	0.4	0.4	0.4	0.4	—	0.4	0.4	0.4	0.4	—	0.4	0.4	0.4	0.4
	Unadjusted	0.4	0.4	0.4	0.4	0.4	0.4	0.4	0.4	0.4	0.4	0.4	0.4	0.4	0.4	0.4
	% Difference	—	–2.1	–2.4	–2.3	–3.4	—	–2.1	–2.4	–2.3	–3.4	—	–2.0	–2.3	–2.3	–3.4
Probability of relapse	Adjusted	—	0.0	0.0	0.0	0.0	—	0.0	0.0	0.0	0.0	—	0.0	0.0	0.0	0.0
	Unadjusted	0.0	0.0	0.0	0.0	0.0	0.0	0.0	0.0	0.0	0.0	0.0	0.0	0.0	0.0	0.0
	% Difference	—	–11.2	–12.6	–12.1	–17.5	—	–14.3	–16.0	–15.4	–21.3	—	–13.6	–15.0	–15.0	–19.7
Treatment effect	Adjusted	—	0.1	0.1	0.1	0.1	—	0.1	0.1	0.1	0.1	—	0.1	0.1	0.1	0.1
	Unadjusted	0.1	0.1	0.1	0.1	0.1	0.1	0.1	0.1	0.1	0.1	0.1	0.1	0.1	0.1	0.1
	% Difference	—	–5.4	–7.9	–7.2	–16.1	—	–5.4	–7.9	–7.2	–16.0	—	–5.4	–7.9	–7.2	–16.0
Width treatment effect CI	Adjusted	—	0.2	0.2	0.2	0.2	—	0.2	0.2	0.2	0.2	—	0.2	0.2	0.2	0.2
	Unadjusted	0.1	0.1	0.1	0.1	0.1	0.1	0.1	0.1	0.1	0.1	0.1	0.1	0.1	0.1	0.1
	% Difference	—	34.1	33.9	34.3	36.5	—	34.1	34.0	34.4	36.5	—	34.1	34.1	34.3	36.5

ENBS, expected net benefit of sampling; EVSI, expected value of sample information; OBF O’Brien-Fleming stopping rule; POC, Pocock stopping rule.

The difference between the adjusted and unadjusted estimates of the primary outcome is greatest for the adaptive designs with 5 analyses compared with 2 analyses and highest for POC compared with OBF. The point estimates are likely to be based on less data, as the interim sample size for early analyses out of 5 is small and the POC stopping boundary is more likely to be crossed at an early interim analysis by design. The adjusted confidence intervals are wider, reflecting the additional uncertainty introduced by the adaptive design.

The differences between the model parameters are small and close to zero for the cost parameters and the utility improvement. The percentage differences are higher for the probability of good response and probability of relapse, reaching 3.42% and 21.29%, respectively. The primary outcome is used to calculate these model parameters and is biased even when there is no correlation between primary and health economic outcomes. The differences for all parameters are greatest for POC5 within each correlation and greatest when the correlation is equal to 0.4.

Overall, the impact of the adjustments for the hypothetical case study is small, with the optimal design changing only when the correlation is 0.4. The adjusted EVSI estimates for the adaptive designs are larger than the unadjusted estimates. The increased EVSI could suggest greater uncertainty as a consequence of the analysis methods used to estimate the adjustments or could reflect the fact that unadjusted approaches underestimate the uncertainty introduced by the adaptive design.

The EVSI values change when there is a change in the decision uncertainty.^12^ If the bias adjustments have little impact on the decision uncertainty, there will be only small differences between the adjusted and unadjusted EVSI estimates, even if there are large differences between the adjusted and unadjusted model parameters estimates. This is illustrated using 2 hypothetical scenarios in the supplementary material.

### Case Study Summary

In the hypothetical case study, we found small differences in the cost of sampling between designs driven by small differences in the ESS. The ENBS was positive and similar for each of the designs, suggesting they were all cost-effective. The effect of the bias adjustment was small and had limited impact on choosing the optimal design. The O’Brien-Fleming stopping rule with 2 analyses had the highest EVSI and ENBS, suggesting this was the most cost-effective design. As the intervention costs were increased and ENBS was recalculated, the potential savings in ESS offered by the adaptive designs gave them a higher ENBS. The O’Brien-Fleming stopping rule with 5 analyses was preferred when variable costs were high, as they offered early interim analyses with a small number of participants and hence a lower ESS and cost of sampling when there was little to be gained in terms of EVSI. The financial benefits of stopping a trial early are likely to be small when the fixed costs are high relative to the variable costs and likewise when the variable costs associated with assessing the trial outcomes in all patients may be high.

## Discussion

We have adapted existing methods of EVSI to guide the design of fixed-sample-size designs to the case of considering adaptive designs. These methods appropriately adjust for the adaptive nature of the design and have been operationalized in the context of a hypothetical case study.

### How This Fits with Existing Literature

We have considered adaptive designs with clinical effectiveness stopping rules based on recommendations by Flight et al.^11^ and suggestions from the public advisory group supporting this research. However, application of VOIA methods could be extended and applied at the interim analysis of an adaptive design to allow research teams to assess the cost-effectiveness of continuing and to inform the design of the rest of the trial. A simple approach would be to update the EVSI calculation with the available data at the interim; however, this does not take account of all possible future interim analyses. Using health economic outcomes during an adaptive trial has been discussed in the literature^33,37–39^; however, care is needed to ensure the preferences of stakeholders are met.^11^

The EcoNomics of Adaptive Clinical Trials (ENACT)^40^ collaboration has explored how the value-based sequential approach of Chick et al.^39^ and Alban et al.^41^ can be applied in the context of publicly funded research in the United Kingdom. Using 2 retrospective case studies, they considered the methodology’s strengths, such as considering the ultimate technology adoption decision in the design and analysis of a trial, and challenges, including the application of the methods within current funding structures.^42,43^

### Implications for Practice and Research

We recommend researchers adjust analyses for the adaptive nature of the designs to avoid introducing bias, reflecting current reporting and regulatory guidance for adaptive designs.^44,45^ As discussed by Flight,^17^ adjusted model parameters cannot always be directly estimated from the trial data, and so alternative methods are required. As such, the difference between adjusted and unadjusted estimates may be a consequence of the different analysis methods as well as biases introduced by the design. We reported the adjusted estimates for the adaptive designs and unadjusted estimates for the fixed-sample-size designs, as we felt that this best reflected the analysis approach that would be undertaken in practice and reflected current guidance.^10^

The VOIA approach outlined offers a formal way to quantify and compare the value of fixed-sample-size and adaptive designs. This will enable researchers to provide a quantified justification for their choice of adaptive design as per the recent guidance from the Food and Drug Administration in the United States.^45^ We anticipate these methods will also be used by research teams to inform discussions on the best choice of trial design.

We have compared the ESS, EVSI, and ENBS to identify the optimal design. Other factors may include the potential maximum sample size. As discussed, the Pocock stopping rule requires a larger sample size if the trial does not stop early compared with the O’Brien-Fleming rule. Funders, for example, will need to consider the financial and practical implications should the trial continue to the maximum sample size.

Following the approach of Willan and Kowgier,^33^ we have assumed the opportunity cost is equal to the a priori INB. However, this will be true only if the new intervention cannot be implemented in practice before the trial ends. The opportunity cost may be zero if the intervention is potentially cost-effective and can be used in practice while research is ongoing.^46^ Research teams should select the appropriate opportunity cost for their setting.

Using this approach may require a large investment of work before the trial is funded. In the CACTUS case study, pilot data and a health economic model were available, reducing the time burden of the VOIA. Application of this approach may be limited to contexts in which an economic model is available or a model can be developed quickly alongside the design of the trial. As highlighted by Flight et al.,^11^ for these methods to be used to their full potential, funding bodies need to consider alternative ways to fund this work.

As with other EVSI methods, the computation time is high.^27,47^ For 5000 PSA samples, it took approximately 5 and 7 h to run the designs with 2 and 5 analyses, respectively. A full range of trial designs should be compared with a high number of PSA samples.^10^ However, this may not be viable given the time constraints associated with designing clinical trials for a grant application. Alternative methods for the calculation of EVSI^47–50^ could decrease the computation time.

We have focused on the commonly used GSD; however, this approach could be considered for other adaptive designs. For example, Ward et al.^51^ used EVSI to compare the optimal design of a 3-arm trial with and without an interim futility analysis.

### Strengths and Limitations

To the best of our knowledge, this is the first adaptation of EVSI to guide the design of a GSD in which interim adaptations are focused on clinical effectiveness. These methods reflect the views of key stakeholders in health technology assessment on the use of health economics in adaptive design^11^ and build on existing guidance and methods in VOIA.^10,12,27^ These methods have the potential to affect the design of adaptive trials that are increasingly used in practice.^5^

We have used a hypothetical case study to illustrate how the methods can be applied in practice. The results are context specific; for example, there were small differences between the ENBS for the designs considered, and the bias adjustments had a limited impact. We cannot draw generalizable conclusions about the performance of adaptive and fixed-sample-size designs. However, the adapted VOIA methods and the presentation of the results can be applied to different contexts.

## Conclusion

Health economics is rarely used in the design and analysis of adaptive clinical trials. We discuss how existing VOIA methods can be adapted to guide the design of a GSD based on the number of analyses and clinical effectiveness stopping rule. This can guide and justify the choice of characteristics and prevent limited research budgets being wasted. We recommend that adjusted analyses are presented to control for the potential impact of the adaptive designs to maintain the accuracy of the calculations.

## Supplemental Material

sj-docx-1-mdm-10.1177_0272989X211045036 – Supplemental material for Expected Value of Sample Information to Guide the Design of Group Sequential Clinical Trials Click here for additional data file. Supplemental material, sj-docx-1-mdm-10.1177_0272989X211045036 for Expected Value of Sample Information to Guide the Design of Group Sequential Clinical Trials by Laura Flight, Steven Julious, Alan Brennan and Susan Todd in Medical Decision Making
