# Supplementary material for: Expected Value of Sample Information to Guide the Design of Group Sequential Clinical Trials
Source: Med Decis Making. 2021 Dec 3;42(4):461–73. doi: 10.1177/0272989X211045036 (PMC9005835; doi:10.1177/0272989X211045036)
Supplement: sj-docx-1-mdm-10.1177_0272989X211045036 – Supplemental material for Expected Value of Sample Information to Guide the Design of Group Sequential Clinical Trials [file sj-docx-1-mdm-10.1177_0272989X211045036.docx]

**Supplementary Material**

1. **Practical Steps for Calculating the Cost of Sampling for an Adaptive Design**

The calculation of the cost of sampling can be more challenging than for the fixed sample size design as the progress of the trial is less predictable, and there is currently limited guidance on how to do this. We propose the following approach which was applied to the hypothetical case study. This approach is based on the maximum required sample size for the chosen design and the proposed recruitment rate. This then gives an estimate of the variable and analysis costs that can be multiplied by the observed sample size and number of analyses for each simulated trial.

Firstly, we divide the study period into four distinct phases and allocate it a specific number of months:

- Set-up - period before first participant is recruited (e.g. 6-months)
- Recruitment – period where participants are recruited into the trial (e.g. 12-months)
- Follow-up – period after last participant recruited but follow-up data collection on-going (e.g. 12-months)
- Trial end – period once all data collected, then analysed and disseminated (e.g. 6-months)

Each cost incurred during the study can be broken down into a monthly cost and allocated to a month in the given phase of the trial. Some costs, such as dissemination costs, may fall into a single month in a single study phase (trial end). Other costs may cover multiple phases, for example, site training might take place predominantly during the trial set-up period but some sites might be trained during the trial recruitment period if the trial continues through interim analyses.

Using the proposed monthly recruitment rate, costs per participant can be converted to a monthly cost. For example, if the anticipated recruitment rate is one participant per centre per month, in a month where six centres are expected to be recruiting the monthly recruitment rate will be six participants. Costs associated with randomising a participant can be multiplied by six to give the monthly cost. All costs are given as a monthly cost so they can be easily combined.

The fixed costs are the sum of the monthly costs in the trial set-up and trial-end phases. The analysis cost is the sum of the analysis costs associated with the analysis of the primary endpoint used for interim decision making.

To calculate the variable costs incurred by all participants the costs incurred by each trial participant each month during the trial recruitment and follow-up periods are added together. This is then added to all other costs incurred during these phases such as staff and meeting costs that continue throughout this period. The sum of these monthly costs is then divided by the maximum number participants to be recruited in the trial to give a cost per participant in each month. The costs in each month of the recruitment and follow-up phases are added together to give a variable cost per participant incurred by all participants during the trial.

To calculate the variable costs incurred by participants in the intervention arm only a similar process is followed. The costs incurred by each trial participant in the intervention arm in each month during the trial recruitment and follow-up periods are added together. These costs are then divided by the maximum number participants to be recruited to the intervention arm. This gives a cost per participant in each month. The costs in the recruitment and follow-up periods are added together to give a variable cost for each participant in the intervention arm during the trial. This is repeated to calculate the variable costs incurred by participants in the control arm including the costs in the recruitment and follow-up phases that are incurred by participants receiving the control arm treatment.

1. **Data Generating Mechanism for Simulating Trials**

A dataset of the expected trial outcomes for individuals randomised to the computer-based intervention arm and usual care control arm of the trial is simulated for each row of the PSA sample. A multivariate distribution that allows the marginal distributions to be non-Normal and where a correction between outcomes can be specified is used. Simulated outcomes include:

1. Percentage of words named correctly measured at baseline, 6 and 9-months follow-up.
2. Resource costs incurred from baseline to 6-months follow-up (not including the costs of delivering the computer-based intervention).
3. EQ-5D score measured at baseline, 6 and 9-months follow-up.
   1. **Correlations**

Correlations between repeated outcomes measured on the same participant at different time points are commonly correlated (53). For example, a patient who has a high percentage of words named correctly at baseline is likely to have a high percentages of words named correctly at 6-months follow-up; a positive correlation. Let $\rho_{t}$ denote the correlation within a given outcome measured at different time points. This correlation is fixed to be 0.5 for all outcomes at each follow-up time point.

Correlation values are varied to explore the effect of correlation between the primary and health economic outcomes on the level of bias in the point estimates. Let $\rho$ denote the correlation. It is assumed that outcomes measured at the same period have correlation $\rho$, for example baseline costs and baseline utility have correlation $\rho$. Outcomes measured at one follow-up time point apart have correlation $\rho^{3}$ and two follow-up time points have correlation $\rho^{4}$. For example, the correlation between baseline costs and utility at 6-months follow-up have correlation $\rho^{3}$. This structure is chosen as if the same correlation value is chosen for all periods these terms cancel out and give a correlation of zero.

At each time point, the correlation between the percentage of words named correctly and utility score is assumed positive as it is feasible that as a person’s word naming ability improves their quality of life and hence their utility score improves. The correlation between the percentage of words named correctly and resource costs is assumed negative as it is feasible that as a person’s word naming ability improves they incur fewer costs as they need less speech and language therapy. The correlation between the utility score and costs is assumed negative as it is feasible that as a person’s quality of life improves they incur fewer costs.

- 1. **Marginal Distributions**

The marginal distributions of simulated parameter values are allowed to be non-Normal and are implemented using copulas (54,55).

A truncated Normal distributions is used to simulate the primary outcomes (percentage of words named correctly) at baseline and follow-up time points. This distribution is truncated between zero and one as is required for percentage outcomes.

EQ-5D utility scores are simulated for baseline and follow-up time points by first simulating disutilities and transforming on to the utility scale by subtracting from one. Simulating disutilities (1 minus the utility) allows utility scores to be negative, representing health states considered to be worse than death but imposing an upper limit of one (full health) (56).

The logNormal distribution represents a small number of patients with high disutility values (low utility values) and the majority of patients with a score lying closer to one. The parameters for the logNormal distribution $(m, s)$ are estimated using

|  | $\left( 1-m \right)=log\left( \frac{{(1-\mu)}^{2}}{\sqrt{\sigma^{2}+{(1-\mu)}^{2}}} \right)$ | (1) |
| --- | --- | --- |
|  | $s=\sqrt{\frac{log(1+\sigma^{2})}{{(1-\mu)}^{2}}}$ | (2) |

where $\mu$ is the mean and $\sigma^{2}$ is the variance.

Resource cost data are simulated using a logNormal distribution to represent the right skew commonly seen, where a small number of participants have high costs (57). The parameters for the logNormal distribution are estimated from the means and standard deviations

|  | $m=log\left( \frac{{(\mu)}^{2}}{\sqrt{\sigma^{2}+{(\mu)}^{2}}} \right)$ | (3) |
| --- | --- | --- |
|  | $s=\sqrt{\frac{log(1+\sigma^{2})}{{(\mu)}^{2}}}$ | (4) |

# Calculating the Cost of Sampling for the Case Study

Financial information from the Big CACTUS grant application was used to inform the cost of sampling for the hypothetical CACTUS case study trial. These costs differ from those in the original Big CACTUS trial. Note here, the calculated costs are based on two-arm scenarios, consider all costs associated with conducting the trial and are estimated based on the Big CACTUS grant application but not the actual costs incurred during the conduct of the Big CACTUS trial.

In contrast to the Big CACTUS trial, all costs associated with conducting the trial were of interest regardless of who incurred these costs. This meant that research costs were considered at 100% of their value, although in the Big CACTUS trial the funder provided 80% of these costs. It was assumed that the costs of usual care provided in the UC and CSLT arms were zero as they would be provided to a participant whether or not they were taking part in the study. The cost of CSLT was considered a trial cost and was included in the cost of sampling. When a trial team calculate the cost of sampling for their setting they will need to consider the most appropriate costs to include depending on the perspective of their analysis.

The components of the cost of sampling for the hypothetical case study are given in Table 1. The fixed costs are £682,415 and are incurred regardless of the design chosen to conduct the trial.

Table 1 Summary of research costs modelled for the case study split by different components

|  | **Description** | **Price** |
| --- | --- | --- |
| **Fixed** | All costs incurred which do not depend on the number of participants and the design of the trial including trial set up costs, dissemination costs and secondary analysis costs | £682,415 |
| **Variable** | Research costs for all study participants including the intervention costs, data collection costs, recruitment costs | £3,371 per participant |
|  | Costs of delivering the computer-based intervention to the intervention arm participants | £769 per participant |
|  | Costs of delivering usual care to the control arm participants during the trial | £0.00 per participant |
| **Analysis** | The cost of one month of a junior statistician’s time to conduct the analysis required at an interim analysis of primary, clinical endpoint | £874 per analysis |
| **Opportunity** | The incremental net benefit taken from R model analysis of the CACTUS pilot data | £2,380 per participant |

# Impact of bias adjustments on EVSI

The EVSI values change when there is a change in the decision uncertainty (18). If the bias adjustments have little impact on the decision uncertainty there will only be small differences between the adjusted and unadjusted EVSI estimates even if there are large differences between the adjusted and unadjusted model parameters estimates. Figure 1 shows two hypothetical scenarios to illustrate when adjustments likely to affect EVSI estimates.

In Figure 1a, the incremental cost and QALY are plotted on a cost-effectiveness plane, with a willingness to pay threshold of £20,000 per QALY represented by the black diagonal line. The red dots represent the PSA results for an adaptive design that have not been adjusted and the blue dots the adjusted PSA results. In this case, the adjustments result in a small reduction in incremental costs and incremental QALY. However, all points lie below the willingness to pay threshold. This suggests there is little decision uncertainty that the new intervention is cost-effective and little value in conducting further research. The adjusted and unadjusted EVSI estimates are unlikely to be affected by the bias adjustments.

The scenario in Figure 1b has much greater decision uncertainty. In the unadjusted case, the new intervention is likely to be more costly and less effective. The adjusted analysis, however, suggests that the new intervention may be more costly but more effective. The unadjusted and adjusted EVSI estimates will differ, with a larger EVSI expected for the unadjusted analysis.


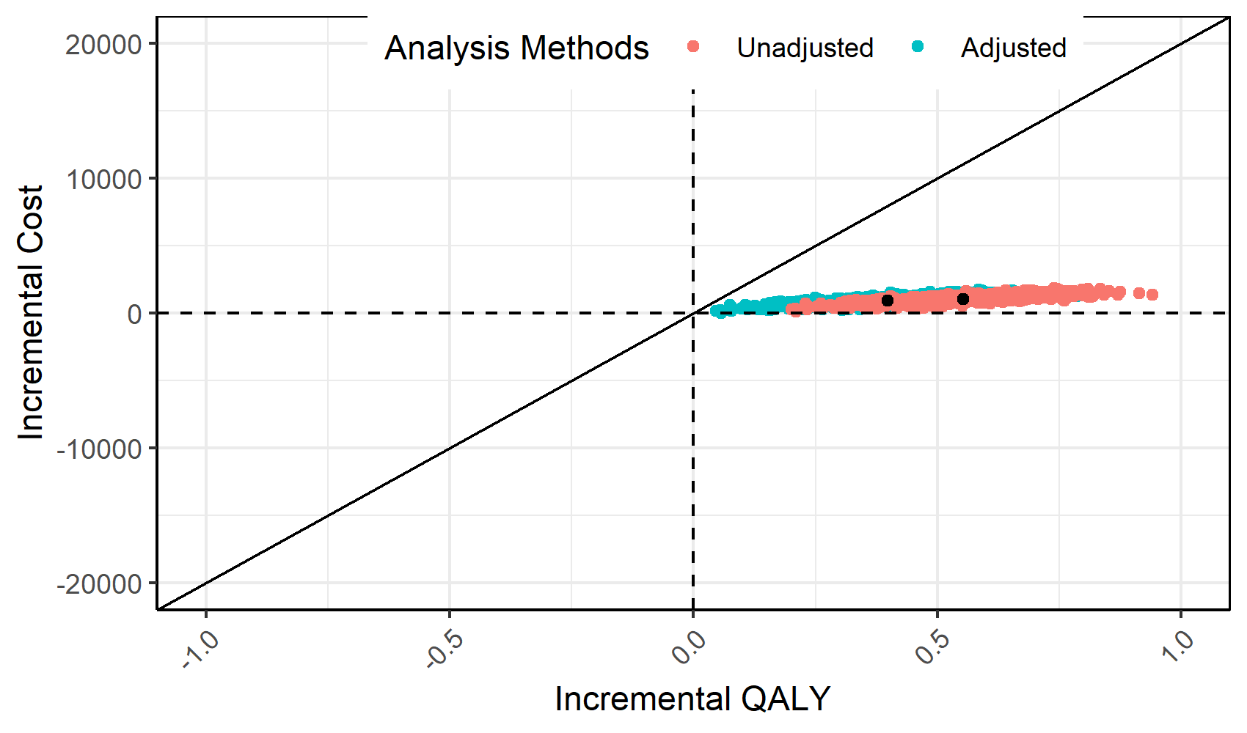


1. Low Decision Uncertainty


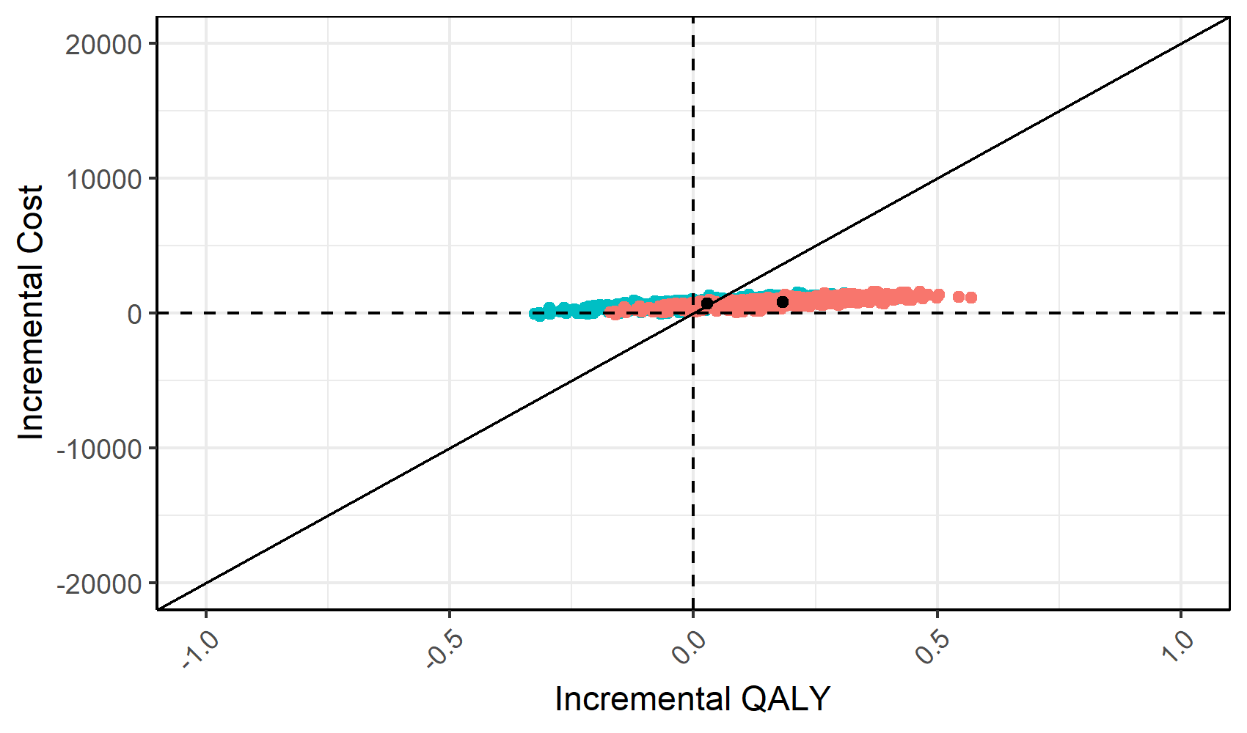


(b) High Decision Uncertainty

Figure 1 illustration of two contrasting scenarios showing how adjustments for the adaptive nature of a clinical trial design are likely to affect the EVSI estimate. The black dots represent the incremental cost-effectiveness ratio

1. **References**

1. National Institute of Health Research. Annual Efficient Studies funding calls for CTU projects [Internet]. 2019 [cited 2021 Mar 31]. Available from: https://www.nihr.ac.uk/documents/ad-hoc-funding-calls-for-ctu-projects/20141

2. Adaptive designs in clinical drug development—an executive summary of the phrma working group. J Biopharm Stat. 2006;16(3):275–83.

3. Bretz F, Koenig F, Brannath W, Glimm E, Posch M. Adaptive designs for confirmatory clinical trials. Stat Med. 2009;28(8):1181–217.

4. Pallmann P, Bedding AW, Choodari-Oskooei B, Dimairo M, Flight L, Hampson L V, et al. Adaptive designs in clinical trials: why use them, and how to run and report them. BMC Med. 2018;16(1):29.

5. Hatfield I, Allison A, Flight L, Julious SA, Dimairo M. Adaptive designs undertaken in clinical research: A review of registered clinical trials. Trials. 2016;17(1).

6. Mistry P, Dunn JA, Marshall A. A literature review of applied adaptive design methodology within the field of oncology in randomised controlled trials and a proposed extension to the consort guidelines. BMC Med Res Methodol. 2017;17(1):108.

7. Bothwell L, Avron J, Khan N, Kesselheim A. Adaptive design clinical trials: A review of the literature and clinicaltrials. gov. BMJ Open. 2018;8(2).

8. RECOVERY Collaborative Group. Dexamethasone in hospitalized patients with Covid-19—preliminary report. N Engl J Med. 2020;

9. Flight L, Arshad F, Barnsley R, Patel K, Julious S, Brennan A, et al. A Review of Clinical Trials With an Adaptive Design and Health Economic Analysis. Value Heal. 2019;22(4).

10. Rothery C, Strong M, Koffijberg HE, Basu A, Ghabri S, Knies S, et al. Value of information analytical methods: report 2 of the ISPOR value of information analysis emerging good practices task force. Value Heal. 2020;23(3):277–86.

11. Flight L, Julious SA, Brennan A, Todd S, Hind D. How can health economics be used in the design and analysis of adaptive clinical trials? A qualitative analysis. Trials. 2020;21(1):1–12.

12. Palmer R, Enderby P, Cooper C, Latimer N, Julious S, Paterson G, et al. Computer therapy compared with usual care for people with long-standing aphasia poststroke: A pilot randomized controlled trial. Stroke. 2012;43(7):1904–11.

13. Whitehead J. The design and analysis of sequential clinical trials. Wiley; 1997.

14. Pocock SJ. Group sequential methods in the design and analysis of clinical trials. Biometrika. 1977;64:191–9.

15. O’Brien PC, Fleming TR. A multiple testing procedure for clinical trials. Biometrics. 1979;35:549–56.

16. Jennison C, Turnbull BW. Group sequential methods with applications to clinical trials. Chapman and Hall/CRC; 2000.

17. Flight L. The use of health economics in the design and analysis of adaptive clinical trials. University of Sheffield; 2020.

18. Fenwick E, Steuten L, Knies S, Ghabri S, Basu A, Murray JF, et al. Value of Information Analysis for Research Decisions—An Introduction: Report 1 of the ISPOR Value of Information Analysis Emerging Good Practices Task Force. Value Heal. 2020;23(2):139–50.

19. Ades AE, Lu G, Claxton K. Expected Value of Sample Information Calculations in Medical Decision Modelling. Med Decis Mak. 2004;24:207.

20. Welton NJ, Ades AE, Caldwell DM, Peters TJ. Research prioritization based on expected value of partial perfect information: A case-study on interventions to increase uptake of breast cancer screening. J R Stat Soc Ser A Stat Soc. 2008;171(4):807–34.

21. Griffin S, Welton NJ, Claxton K. Exploring the Research Design Space: The Expected Value of Information for Sequential Research Designs. Med Decis Mak. 2010;30:155.

22. Boyd KA, Fenwick E, Briggs A. Using an iterative approach to economic evaluation in the drug development process. Drug Dev Res. 2010;71(8):470–7.

23. Flight L, Julious SA. Practical guide to sample size calculations: An introduction. Pharm Stat. 2016;15(1).

24. Whitehead J. On the bias of maximum likelihood estimation following a sequential test. Biometrika. 1986;73(3):573–81.

25. Whitehead J. Supplementary analysis at the conclusion of a sequential clinical trial. Biometrics. 1986;461–71.

26. Emerson SS, Fleming TR. Parameter estimation following group sequential hypothesis testing. Biometrika. 1990;77(4):875–92.

27. Strong M, Oakley JE, Brennan A, Breeze P. Estimating the expected value of sample information using the probabilistic sensitivity analysis sample: A fast, nonparametric regression-based method. Med Decis Mak. 2015;35(5):570–83.

28. Welton NJ, Madan JJ, Caldwell DM, Peters TJ, Ades a E. Expected Value of Sample Information for Multi-Arm Cluster Randomized Trials with Binary Outcomes. Med Decis Mak [Online] [Internet]. 2013;34(April):352–65. Available from: http://mdm.sagepub.com/

29. Heath A, Kunst NR, Jackson C, Strong M, Alarid-Escudero F, Goldhaber-Fiebert JD, et al. Calculating the Expected Value of Sample Information in Practice: Considerations from Three Case Studies. Med Decis Mak. 2020;40(3).

30. Kunst NR, Wilson E, Alarid-Escudero F, Baio G, Brennan A, Fairley M, et al. Computing the Expected Value of Sample Information Efficiently: Expertise and Skills Required for Four Model-Based Methods. value i. 2020;23(6):734–42.

31. Eckermann SD, Willan AR. GLOBALLY OPTIMAL TRIAL DESIGN FOR LOCAL DECISION MAKING. Health Econ. 2009;18:203–16.

32. Zhao G, Chen W. Enhancing R&D in science-based industry: An optimal stopping model for drug discovery. Int J Proj Manag [Internet]. 2009;27(8):754–64. Available from: http://dx.doi.org/10.1016/j.ijproman.2009.01.003

33. Willan AR, Kowgier M. Determining optimal sample sizes for multi-stage randomized clinical trials using value of information methods. Clin Trials. 2008;5:289–300.

34. Latimer NR, Dixon S, Palmer R. Cost-Utility of Self-Managed Computer Therapy for People With Aphasia. Int J Technol Assess Health Care [Internet]. 2013;29(04):402–9. Available from: http://www.journals.cambridge.org/abstract_S0266462313000421

35. Palmer R, Dimairo M, Latimer N, Cross E, Brady M, Enderby P, et al. Computerised speech and language therapy or attention control added to usual care for people with long-term post-stroke aphasia: the Big CACTUS three-arm RCT. Health Technol Assess. 2020;24(19):1.

36. National Institute for Health and Care Excellence. Guide to the Methods of Technology Appraisal [Internet]. 2013 [cited 2017 Jul 12]. Available from: http://www.nice.org.uk/article/pmg9/chapter/foreword

37. Berry DA, Ho C-H. One-sided sequential stopping boundaries for clinical trials: A decision-theoretic approach. Biometrics. 1988;219–27.

38. Pertile P, Forster M, Torre D La. Optimal Bayesian sequential sampling rules for the economic evaluation of health technologies. J R Stat Soc Ser A Stat Soc. 2014;177(2):419–38.

39. Chick S, Forster M, Pertile P. A Bayesian decision theoretic model of sequential experimentation with delayed response. J R Stat Soc Ser B (Statistical Methodol. 2017;79(5):1439–62.

40. EcoNomics of Adaptive Clinical Trials (ENACT) [Internet]. Value-adaptive Clinical Trial Designs for Efficient Delivery of NIHR Research. 2021 [cited 2021 Mar 24]. Available from: https://www.sheffield.ac.uk/scharr/research/centres/ctru/enact

41. Alban A, Chick S, Forster M. Value-based clinical trials: selecting trial lengths and recruitment rates in different regulatory contexts. Discuss Pap 20/01, Dep Econ Univ York [Internet]. 2020; Available from: https://ideas.repec.org/p/yor/yorken/20-01.html

42. Flight L, Brennan A, Chick S, Forster M, Julious SA, Tharmanathan P. Value-Adaptive Clinical Trial Designs for Efficient Delivery of Research – Actions, Opportunities and Challenges for Publicly Funded Trials. 2021.

43. Forster M, Flight L, Corbacho B, Keding A, Ronaldson S, Tharmanathan P, et al. Application of a Bayesian Value-Based Sequential Model of a Clinical Trial to the HERO and CACTUS Case Studies. 2021.

44. Dimairo M, Pallmann P, Wason J, Todd S, Jaki T, Julious SA, et al. The Adaptive designs CONSORT Extension (ACE) Statement: a checklist with explanation and elaboration guideline for reporting randomised trials that use an adaptive design. BMJ. 2020;17:369.

45. U.S. Food and Drug Administration. Guidance for Industry: Adaptive Design Clinical Trials for Drugs and Biologics. [Internet]. 2019 [cited 2020 Mar 26]. Available from: http://www.fda.gov/downloads/Drugs/Guidances/ucm201790.pdf

46. Heath A, Manolopoulou I, Baio G. Efficient Monte Carlo estimation of the expected value of sample information using moment matching. Med Decis Mak. 2018;38(2):163–73.

47. Menzies NA. An efficient estimator for the expected value of sample information. Med Decis Mak. 2016;36(3):308–20.

48. Jalal H, Goldhaber-Fiebert JD, Kuntz KM. Computing expected value of partial sample information from probabilistic sensitivity analysis using linear regression metamodeling. Med Decis Mak. 2015;35(5):584–95.

49. Jalal H, Alarid-Escudero F. A Gaussian approximation approach for value of information analysis. Med Decis Mak. 2018;38(2):174–88.

50. Ward M, Grayling M, Wason J, Welton NJ, Haji-Ali AL, Jalal H, et al. PSU4 VALUE of Information for Adaptive Trials: Proof of Concept Study in MULTI-Arm MULTI-STAGE Trials of Interventions for the Prevention of Surgical Site Infections. In: ISPOR Europe 2020. Value in Health; 2020. p. S738.

51. Claxton K, Palmer S, Longworth L. Informing a decision framework for when NICE should recommend the use of health technologies only in the context of an appropriately designed programme of evidence development. Health Technol Assess (Rockv). 2012;16(46):1–323.

52. Faria R, Gomes M, Epstein D, White IR. A guide to handling missing data in cost-effectiveness analysis conducted within randomised controlled trials. Pharmacoeconomics. 2014;32(12):1157–70.

53. Walters SJ, Jacques RM, Henriques-Cadby IB dos A, Candlish J, Totton N. Sample size estimation for randomised controlled trials with repeated assessment of patient-reported outcomes: What correlation between baseline and follow-up outcomes should we assume? Trials. 2019;20(1):1–16.

54. Nelsen RB. An introduction to copulas. Springer Science & Business Media; 2007.

55. Hofert M, Mächler M. Nested archimedean copulas meet R: The nacopula package. J Stat Softw. 2011;39(9):1–20.

56. Patrick DL, Starks HE, Cain KC, Uhlmann RF, Pearlman RA. Measuring preferences for health states worse than death. Med Decis Mak. 1994;14(1):9–18.

57. Briggs A, Claxton K, Schulpher M. Decision Modelling for Health Economic Evaluation. Oxford: Oxford University Press; 2006.
